# An overview of smoking practices in Pakistan

**DOI:** 10.12669/pjms.312.6816

**Published:** 2015

**Authors:** Noreen Shah, Saad Siddiqui

**Affiliations:** 1 Dr. Noreen Shah, MBBS, MAMS (Austria), Senior Lecturer, Department of Community Medicine, Khyber Girls Medical College, Peshawar, Pakistan; 2 Dr. Saad Siddiqui, MBBS (AKU), Resident, Department of Radiology, The Aga Khan University, Karachi, Pakistan

**Keywords:** Smoking, Tobacco, Adolescents, Pakistan, Shisha

## Abstract

Smoking remains a major player in morbidity and mortality worldwide. It is a matter of immense public health importance as single leading cause of preventable deaths. The aim of this study was to assess smoking practices that prevail across Pakistan & Attitude of people towards this issue. We conducted an extensive search on major databases as well as search of bibliography of published literature for studies assessing Attitudes and Practices of tobacco smoking that prevail across Pakistan. Data from available studies was abstracted and utilized in preparation of this manuscript. After screening of 613 articles, we were able to identify 22 studies matching our criteria for inclusion.

Majority of studies reported adolescence as time of initiation. Average national prevalence was 21.6%. A significant portion of smokers comprised of females. The prevalence of smoking in healthcare professionals ranged from 32 – 37%. Passive smoking was a major contributor of tobacco exposure. Prevalence of ‘Shisha’ use was 33%. Smoking continues to be a major Public Health issue in Pakistan. The prevalence in healthcare professionals and adolescents is alarming. Adequate measures need to be taken to ensure its control.

## INTRODUCTION

Smoking is the practice of combustion and inhalation of tobacco (or other recreational drugs). Its history can be traced back to 5000 BC. It is one of the major public health challenges in today’s world and single most important cause of preventable deaths worldwide.^1^

Smoking is a major risk factor for non-communicable diseases like Acute Coronary Syndrome, Cerebrovascular accidents, debilitating chronic diseases like atherosclerosis, hypertension and Chronic Obstructive Pulmonary Disease (COPD). It is a leading cause of malignancies including cancers of Lung, oral cavity, larynx, pancreas and Transitional cell carcinoma of urothelium.^2^

Cigarettes sold in developing countries usually have low quality and a high tar content making them even more injurious to health. According to World Health Organization (WHO) there were 5.4 million tobacco related deaths worldwide in 2004.^3^,^4^ Smoking also decreases immunity and hence ability to fight off diseases, making smokers more susceptible to opportunistic infections. Common communicable illnesses like viral flu usually get complicated in smokers due to the same reason. This fact has importance in smokers undergoing surgical procedures as they suffer from poor post-operative wound healing and increased chance of superficial surgical site infections (SSSI).^5^,^6^

The purpose of this study was to have an insight into smoking practices that prevail across Pakistan & the Attitude of people towards this Public Health issue.

## METHODS

We conducted an extensive literature search using Index Medicus/Medline, PakMediNet and Google Scholar as search engines. We also included studies by searching bibliography of available literature. The search included articles assessing knowledge, attitude and practices of people regarding smoking in Pakistan. We included studies from all strata of population (adolescents, rural population, women, healthcare workers etc.) and studies in all languages. All studies assessing knowledge about smoking and its hazards & Practices regarding smoking were included for gathering data. Studies mentioning use of other recreational drugs (e.g. marijuana, cocaine, heroin etc.) were excluded from final data gathering process.

A total of 613 studies were found upon literature search. After initial screening of abstracts 66 were identified as potential for inclusion and after final literature search by going through manuscripts, 22 studies were finally selected for inclusion and abstraction.

## RESULTS

**Fig.1:**
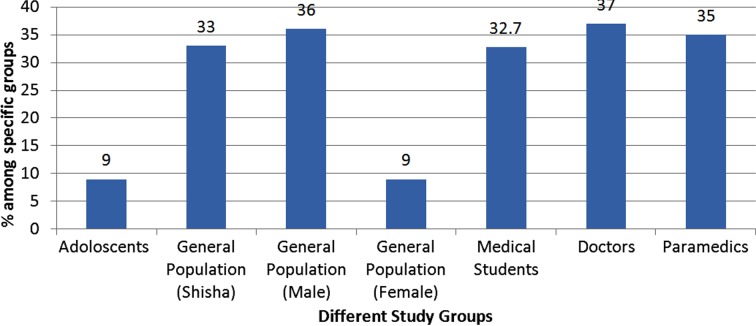
Relative percentage of Active smokers in different study populations.

Majority of studies reported adolescence as time of initiation of smoking with one particularly giving the figure of 17 ± 2.7 years as time of initiation. Overall prevalence of smoking reported by different studies ranged from 16.7 – 33% (Average national figure 21.6% with 36% males and 9% females). In a study conducted in rural Pakistan 10% of females agreed to be active smokers. The prevalence of water pipe smoking or *‘Shisha’* was also found to be 33%. (Fig.1)

Majority of the studies had medical students as their participants followed by healthcare professional including doctors. The prevalence of smoking in this population ranged from 32.7 to 37%. Similarly in a data from a single centre 35% of paramedics agreed to be active smokers.

Results from studies assessing reasons for start or continuation of smoking showed stress relief, perception of image, companionship, leisurely independence, and as sign of masculinity to major contributors. (Table-I)

**Table I:** Reasons for Smoking among Male subjects (Mubeen et al. 2011)^25^

Reasons for Smoking	%
Stress Relief	74
Image Perception	62
Companionship/Peer Pressure	54
Leisurely independence	46
Sign of Masculinity	44

About 9% of adolescents agreed to be active smokers with majority of them reporting to be exposed to passive smoking.11.7% of commuters in air conditioned coaches admitted that they smoked while travelling exposing fellow commuters to passive smoking.

In a major survey done among females, very few had idea that smoking can lead to cardiovascular and obstetrical complications. However anti-smoking messages demonstrating complications of smoking were reported to be effective for smoking cessation.

## DISCUSSION

Smoking remains a major public health issue in Pakistan.^7^,^8^ It is one of the leading risk factor for a number of illnesses including lung and other malignancies. It has been reported that up to 87% cases of lung carcinoma are detected in smokers. Smoking generally increases risk of developing lung carcinoma by 10 fold and people with 40 or more pack years history may have up to 60 times increased risk of developing lung cancer.^9^-^11^

From our search we found smoking to be predominantly more prevalent among males in this region however a significant number of females also reported to be active smokers.^12^ This includes data from both rural and urban populations.^13^ Results from study done by Bhanji et al. demonstrated that although aware of pulmonary complications, majority of female smokers did not have knowledge about cardiovascular & obstetrical complications arising as a result of smoking (e.g. low birth weight, premature birth, congenital anomalies and stillbirths).^14^

Adolescents were found to be the most vulnerable population to pick up habit of smoking. Results from various surveys conducted on this age group demonstrated that 9 – 14% of school children were regularly involved in this habit.^11^,^15^,^16^

The prevalence of smoking in medical students, doctors and healthcare professionals ranged from 32 – 37% as reported by multiple studies. These figures are alarming in way as healthcare professionals are perceived to be the ones enforcing initiatives for smoking control and cessation.^11^,^17^-^19^

Apart from cigarette smoking, the trend of *‘Shisha’* (water pipe) smoking is also on the rise in Pakistan. It is perceived to be more socially acceptability and is often linked to culture. Contrary to popular belief, *‘Shisha’* smoking is more injurious to health compared to cigarettes as it lacks appropriate filters, resulting in inhalation of toxins in excessive amounts. Akl et al. reported prevalence of *‘Shisha’* use in Pakistan to be 33%.^11^,^20^,^21^

Passive smoking is also one of the major contributors to lung diseases. Published data states that every year 3000 nonsmokers succumb to complications of passive smoking.^9^ Anti-smoking laws for public areas have only recently come into the limelight, and even they are not being properly implemented. In a survey conducted by Mal et al. 11.7% commuters of air conditioned public transport reported smoking while travelling and while exposing other passengers to the cigarette smoke.^22^ Similarly study by Khuwaja et al. reported that majority of adolescents were exposed to passive smoking mostly by father smoking at home.^15^

Advertisements have proven to be a major culprit in promoting smoking. Only recently, laws have been introduced in Pakistan against such advertisement strategies. A study conducted by Zaidi et al. demonstrated that displaying pictures of complications of smoking (e.g. patient with oral cancer, those using electronic voice box and patients on ventilators for respiratory support) on cigarette packs were deemed effective in curbing this habit among high school students.^23^

Even though it has been estimated that by increasing the price of cigarettes by just 10%, an immediate decrease of 4.8% and a long term decrease of 11.7% in cigarette use can be achieved, the economics associated with the industry are proving to be major hindrance to tobacco control. Not only does this industry contribute millions of rupees annually to the national exchequer in the form of taxes, being a major cash crop tobacco is also major source of income for scores of individuals in this region.^11^,^24^

## CONCLUSION

Smoking continues to be a major Public Health issue in Pakistan. The prevalence in adolescents and healthcare professionals is alarming. Adequate measures like anti-smoking education need to be in place to control this problem.
